# Notice of Three Children at a Birth

**Published:** 1844-11-30

**Authors:** Robert Annan

**Affiliations:** Surgeon, Kinross


### NOTICE OF THREE CHILDREN AT A BIRTH.

BY ROBERT ANNAN, ESQ., SURGEON, KINROSS.

Mrs. S., robust, of ordinary height, the wife of a manufacturer in Kinross, aged 38, the mother of seven children, was taken in labour, about 5 p. m., on the 16th of April, 1844. From her uncommon size, and having had twins at her previous confinement, the labour had been looked forward to with some anxiety. About 12 p. m., she was delivered of a female child, of full size. A ligature being passed on the cord (and the mother fully anticipating a second child,) the head of another, and feet of a third child were found presenting. As the head had advanced considerably, no time was lost in pushing back the feet, and in rather less than fifteen minutes a boy, a little less in size than the first, was born. The third, a girl of full size, followed (footling) in about twenty minutes. The placenta (which I regret was not preserved,) soon came away, in one mass, the uterine membranes being common to the whole; although, from the appearances presented, I doubt not that injection would have shown little, if any communication among the placental vessels of the three children. The mother has been able to nurse one of the children. The three are now alive and well.

In many systematic works, it is stated that plurality of children, in utero, cannot be positively ascertained previous to actual labour. In the present case, the mother had, for weeks previous, no doubt on the subject; and in another case, which I attended about six years ago, the mother, an intelligent individual, minutely described the separate movements of twin children, at least two months previous to the birth.—*London and Edin. Monthly Journal.*

